# Changes in the Biomarkers of Oxidative/Nitrosative Stress and Endothelial Dysfunction Are Associated with Cardiovascular Risk in Periodontitis Patients

**DOI:** 10.3390/cimb43020051

**Published:** 2021-07-15

**Authors:** Nadia Ferlazzo, Monica Currò, Gaetano Isola, Silvia Maggio, Maria Paola Bertuccio, Angela Trovato-Salinaro, Giovanni Matarese, Angela Alibrandi, Daniela Caccamo, Riccardo Ientile

**Affiliations:** 1 Department of Biomedical and Dental Sciences and Morpho-Functional Imaging, University of Messina, 98123 Messina, Italy; silviamaggio2@gmail.com (S.M.); mp.bertuccio@gmail.com (M.P.B.); gmatarese@unime.it (G.M.); dcaccamo@unime.it (D.C.); ientile@unime.it (R.I.); 2 Department of General Surgery and Surgical-Medical Specialties, School of Dentistry, University of Catania, Via S. Sofia 78, 95123 Catania, Italy; gaetano.isola@unict.it; 3 Department of Biomedical Sciences, University of Catania, 95123 Catania, Italy; trovato@unict.it; 4 Unit of Statistical and Mathematical Sciences, Department of Economics, University of Messina, 98123 Messina, Italy; angela.alibrandi@unime.it

**Keywords:** 3-nitrotyrosine (NT), asymmetric dimethylarginine (ADMA), cardiovascular disease, coenzyme Q10 (CoQ10), inflammasome, peripheral blood mononuclear cells (PBMC), periodontitis (PT)

## Abstract

Patients with cardiovascular disease (CVD) and periodontitis (PT) show shared risk factors as result of the altered molecular mechanisms associated with pathological conditions. The aim of our study was to evaluate if the plasma biomarkers associated with endothelial dysfunction may also be related to alterations in the inflammatory status in peripheral blood mononuclear cells (PBMC). Patients with PT, coronary heart disease (CHD), or both diseases as well as controls were enrolled. Plasma levels of coenzyme Q10 (CoQ10), 3-nitrotyrosine (NT), and asymmetric dimethylarginine (ADMA) were assessed using HPLC. mRNA levels of caspase-1 (*CASP1*), NLR family pyrin domain containing 3 (*NLRP3*), and tumor necrosis factor-α (*TNF-α*) in PBMC from the recruited subjects were quantified using real-time PCR. Patients with PT + CHD showed lower CoQ10 plasma levels and increased concentrations of NT in comparison to healthy subjects. ADMA levels were higher in CHD and PT + CHD patients compared to controls. Transcript levels of *CASP1*, *NLRP3*, and *TNF-α* were up-regulated in PBMC from all patient groups when compared to healthy subjects. Our results suggest a possible causal link between oxidative stress, high levels of NT and ADMA, and inflammasome activation, which may be involved in the endothelial inflammatory dysfunction leading to the pathogenesis and progression of CHD in PT patients.

## 1. Introduction

Cardiovascular disease (CVD) includes various diseases that affect cardiac muscle and the vessels associated with it, such as angina, myocardial infarction, and stroke [1,2]. A chronic exposure to oral bacteria or their toxins causing periodontitis (PT) can generate pathological alterations affecting the vessel wall and can act as a precursor to atherosclerosis in susceptible patients [3]. Experimental evidence has shown that monocytes and endothelial cells can be stimulated by several pathogens to produce pro-inflammatory cytokines, with consequent plasma increase in fibrinogen, C-reactive protein (CRP), haptoglobin, α-1 anti-trypsin, and other components of the APR (acute phase response) that lead to an increased risk of thrombogenic phenomena [4]. A condition of chronic inflammation, such as that associated with CVD and PT, consistently increases the production of reactive oxygen species (ROS) and reactive nitrogen species (RNS), which is usually kept under control by antioxidants in order to prevent cell damage [5,6]. In particular, the concentration of these oxidants becomes elevated as a result of the increased production by the cells of the innate immune system, such as neutrophils and macrophages, and the occurring imbalance between antioxidants and ROS/RNS [6,7]. These events ultimately lead to oxidative stress, negatively affecting tissue integrity through the oxidation of various biochemical structures, such as DNA, lipids, and proteins [5,6].

Recently, a direct and common thread between PT and CVD has been suggested since people with PT have a higher risk of a cardiovascular event (stroke, heart attack etc.) that is two to three times higher than average. Thus, it seems likely that a cluster of shared biochemical alterations leading to PT and systemic inflammation is also responsible for CVD development [8].

In patients who are affected by CVD or periodontitis, the condition of low Coenzyme Q10 (CoQ10) levels has been reported [9]. This compound is a cofactor, known also as ubiquinone, that is involved in the production of ATP in the mitochondrial respiratory chain. It takes part in redox reactions and plays a role as an antioxidant by reducing ROS [10].

Epidemiological studies have also shown a correlation between CVD and levels of 3-nitrotyrosine (NT), a marker of nitrosative stress, derived from the nitration process of the tyrosine residues in plasma protein and in atherosclerotic lesions, which has been found to increase in several inflammatory conditions, including PT [11,12]. Asymmetric dimethylarginine (ADMA) is an endogenous inhibitor of nitric oxide (NO) synthase, promoting enzyme uncoupling. This, in turn, leads to peroxynitrite production and nitrosative stress in the vascular endothelial and smooth muscle cells [13]. We recently demonstrated that patients with coronary heart disease (CHD) and PT (PT + CHD) presented higher levels of serum ADMA compared to healthy subjects and PT patients. Moreover, a positive correlation between ADMA and CRP levels was found [14].

Oxidative and nitrosative stress can facilitate both disease onset and/or progression by evoking immune responses resulting in a higher expression of pro-inflammatory cytokines or driving NLRP3 (NLR family pyrin domain containing 3) inflammasome activation [15,16]. Inflammasomes are a group of cytosolic multiprotein complexes that represent a major innate immune response platform, promoting the secretion of inflammatory cytokines, and is likely to control inflammation in the development of CVD [17].

In this study, we aimed to extend the impact of ADMA, NT, and CoQ10 in patients with PT, coronary heart disease (CHD), or both (PT + CHD) in order to characterize their possible role as predictive additional biomarkers in subjects affected by both pathological conditions. In parallel, to evaluate the inflammatory status of these conditions, we analyzed the transcription levels of inflammasome-associated genes in peripheral blood mononuclear cells (PBMC) from the recruited subjects.

## 2. Materials and Methods

Patients with PT (N = 15), CHD (N = 15), or both diseases (PT + CHD) (N = 14) and healthy controls (N = 19) matched for age and sex were selected among those who attended the Department of Periodontology, School of Dentistry, or the Department of Cardiology of the University of Messina, Messina, Italy from May 2018 to April 2020. All patients gave written informed consent; ethical approval was obtained from the committee of the University of Messina (#12-16, 22/03/2016), and the experimental procedures applied in humans were in accordance with the provisions of the World Medical Association’s Declaration of Helsinki, as revised 2016 for medical research. The inclusion and exclusion criteria for PT and CHD were reported in [14].

The clinical and medical characteristics (sex, age, body mass index, hypertension, diabetes, dyslipidaemia, previous CVD events) and medications were assessed in all enrolled subjects. The presence of diabetes mellitus was based on the history of the patient or a fasting blood glucose ≥126 mg/dL. Body Mass Index (BMI) was estimated using the weight of the patient divided by the square of the patient’s height, i.e., kg/m^2^.

The periodontal evaluation comprised probing depth (PD), clinical attachment loss (CAL), bleeding on probing (BOP), and plaque score (PI). CAL was recorded as PD plus recession at the cemento–enamel junction as a reference for CAL measurements. All clinical periodontal parameters were recorded at six sites per tooth on all teeth present excluding third molars.

Duplicate full-mouth periodontal evaluations were performed by two calibrated examiners who were not involved in the subsequent data analysis using a manual periodontal probe (UNC-15, Hu-Friedy, Chicago, IL, USA). In case of discordant measurements of ≥2 mm PD, a new clinical assessment was performed. Intra- and inter-examiner reproducibility of CAL was assessed from randomly selected patients. The inter-examiner reliability test resulted in an agreement of 85.3% (k = 0.62) for the primary outcome (CAL). The intra-examiner agreement was evaluated by the measurement of Cohen’s k coefficient, which was 0.856, which equalled a high degree of reliability. The kappa coefficient was also calculated for the measurements taken during each follow-up session and an acceptable degree of reliability (ICC = 0.806) was established for every examination.

### 2.1. Measurement of CoQ10, Nitrotyrosine and ADMA Concentrations

Blood samples were collected from all subjects, cooled on ice immediately, and centrifuged at 4 °C (800× *g* per 10 min). Samples were stored at −20 °C until analysis.

Levels of CoQ10, nitrotyrosine and ADMA were assessed by the use of commercially available kits for high performance liquid chromatography (HPLC) measurements (Eureka, Ancona, Italy). In fasting conditions, reference ranges were <830 µg/L for CoQ10, <100 µg/L for nitrotyrosine and 0.3–1.3 µM for ADMA.

Levels of hs-CRP were assessed by a commercially available nephelometric assay. An hs-CRP level higher than 3 mg/L is associated with increased risk of CVD. Plasma lipids were determined by routine methods.

### 2.2. PBMC Collection

PBMC were isolated by centrifugation on a Ficoll-Histopaque density gradient. The blood collected in test tubes containing EDTA was diluted in a ratio of 1:2 in phosphate buffer (PBS), layered on 4 mL of Ficoll and centrifuged at 400× *g* for 20 min. PBMC, layered in the Ficoll–plasma interface, were harvested, washed twice with PBS, and stored at −80 °C until use.

### 2.3. Quantitative Studies of Gene Expression in PBMC

Total RNA isolation from PBMC was carried out using TRIzol reagent (Invitrogen, Milan, Italy) according to the manufacturer’s instructions. Two micrograms of total RNA were reverse transcribed into cDNA by using the High-Capacity cDNA Archive Kit. The TNF-α and IL-1β mRNA levels were quantified by SYBR green-based real-time PCR. β-Actin was used as an endogenous control. The primer sequences used are listed in Table 1. Quantitative PCR reactions were carried out in 10 µL reactions containing 1x SYBR green PCR Mastermix, 0.1 µM specific primers, and 25 ng RNA converted into cDNA. Real-time PCR was performed in a 7900 HT Fast Real-Time PCR System (Applied Biosystems, Foster City, CA, USA) with the following profile: one cycle at 95 °C for 10 min, followed by 40 cycles at 95 °C for 15 s, and 60 °C for 1 min. For SYBR green assays, a standard dissociation stage was added to assess primer specificity. Data were collected with SDS 2.3 software (Applied Biosystems, Foster City, CA, USA) and analyzed using the 2^−ΔΔCt^ relative quantification method.

### 2.4. Power and Sample Size Analysis

Sample size calculation was based on the primary outcome, which was represented by ADMA levels. Assuming an effect size of 0.45 (as results by literature, Guerrero 2014), an a priori power of 0.80, an alpha level probability of 0.05, and a number of groups equal to 4, the sample size calculation (performed by means of F test, one-way ANOVA with fixed effects) suggested that a minimum number of 60 subjects were needed.

### 2.5. Statistical Analysis

The numerical data were expressed as mean and standard deviations, and the categorical variables were expressed as number and percentage. Most of the examined variables were normally distributed, as verified by Kolmogorov–Smirnov test; consequently, the parametric approach was used.

For each marker (CoQ10, NT, ADMA levels) and for each numerical variable, statistical comparisons between the four groups (PT, CHD, PT + CHD and CTR) were performed applying One Way ANOVA (with fixed effects) followed by post hoc comparisons using the HSD Tukey test.

The chi-square test was applied in order to evaluate possible differences among the four groups with reference to categorical variables (such as sex and education).

The Pearson correlation test was applied in order to assess the existence of any significant interdependence between the examined variables, both for whole casuistry and for each group of patients.

A receiver operating characteristic (ROC) curve was realized in order to determine the optimal cut-off of the examined markers (CoQ10, NT and ADMA levels) and to discriminate between patients and control subjects; the area under the curve (AUROC) was calculated with a relative 95% confidence interval and statistical significance. Sensitivity, specificity, negative predictive value (NPV), positive predictive value (PPV), and diagnostic accuracy (DA) were evaluated for the cut-off of 870 (related to CoQ10), 88 (related to NT), and 1.25 (related to ADMA), resulting through ROC analysis.

Finally, univariate and multivariate linear regression models were estimated in order to identify significant predictive factors of CoQ10, NT, and ADMA levels; the tested covariates were: age, gender, education, PT, CHD, periodontal parameters (number of teeth, Cal, PD, BOP and Plaque index), PCR, fasting glucose, and VES. For the estimation of the multivariate model, the stepwise procedure (backward elimination) was used. In each model, we report, the B coefficient, the relative 95% confidence interval (C.I.), and the *p*-value for each variable.

Statistical analyses were performed using the SPSS 22.0 for Window package.

## 3. Results

The anthropometric features and biochemical parameters of the recruited subjects are summarized in Table 2. There were no significant differences in age, sex, BMI, or in prevalence in the number of smokers between the groups. In comparison to PT and healthy subjects, patients in the CHD and PT + CHD groups had previous CVD events (atrial fibrillation, angina pectoris, stroke, heart failure) and took CVD drugs (antihypertensive, statins, low-dose aspirin, beta blockers). Patients with PT, CHD, and PT + CHD showed increased values of CRP and VES in comparison to healthy subjects.

Dental variables in patients and controls are shown in Table 3. The number of teeth present and the values of the periodontal parameters (clinical attachment level, CAL; probing depth, PD; bleeding on probing, BOP; plaque index, PI) were significantly higher in patients with PT, CHD, and PT + CHD compared to control subjects (*p* < 0.001). Moreover, the periodontal parameters were significantly higher in patients in the PT and PT + CHD groups compared to patients with CHD.

**Figure 1:**
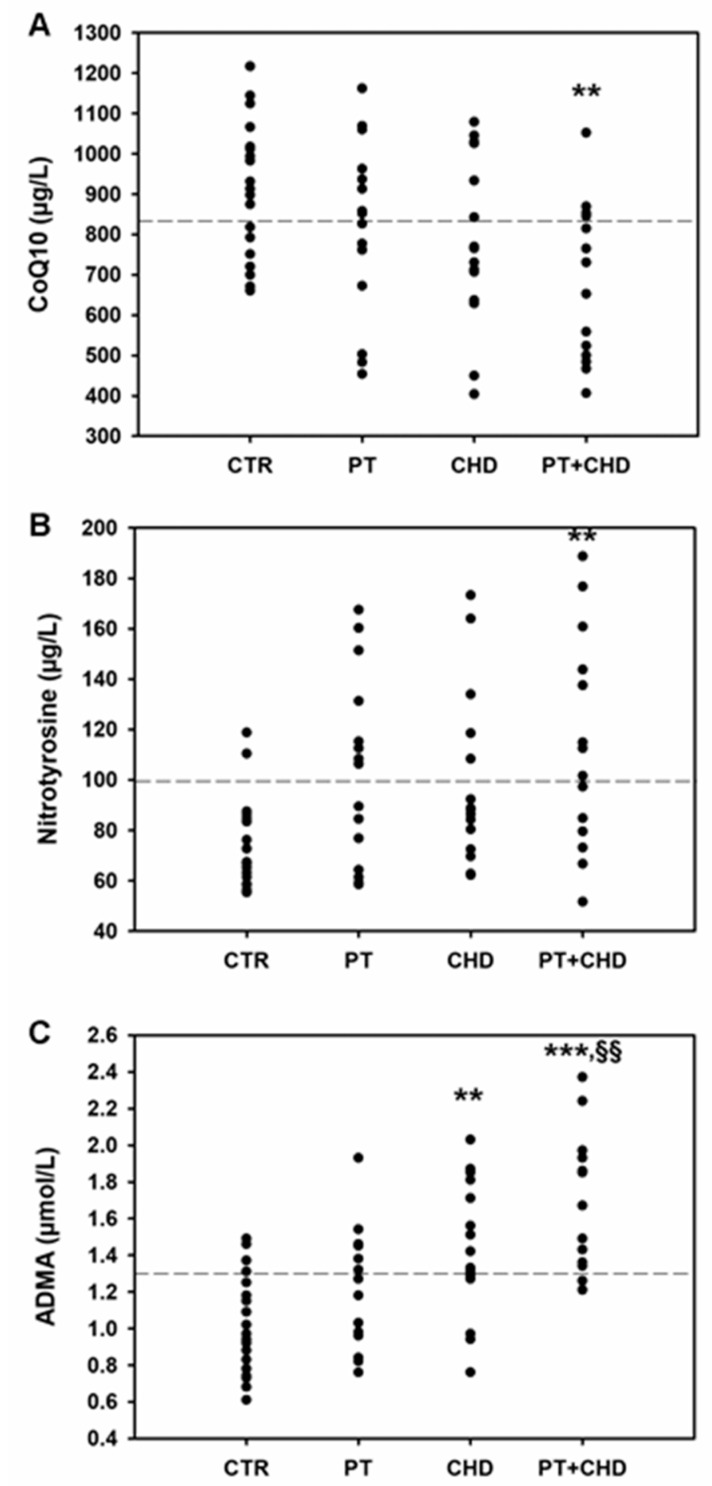
Values of CoQ10 (**A**), NT (**B**) and ADMA (**C**) in patients and control subjects. ** *p* < 0.01 and *** *p* < 0.001 significant differences vs control subjects; §§ *p* < 0.01 significant difference vs PT patients. Dotted lines indicate the limit of reference values.

The mean value of plasma CoQ10 were only in the normal range in control subjects, while patients showed a reduction of CoQ10, which reached the significantly lowest values in the PT + CHD patients (*p* = 0.008, Figure 1A).

The mean concentration of plasma NT were lower in control subjects compared to patients. No significant differences have been observed between PT and CHD patients vs controls, while the NT concentrations in PT + CHD patients were significantly increased (*p* = 0.005) compared to those of healthy subjects (Figure 1B). 

ADMA levels were significantly higher in CHD (*p* = 0.003) and PT + CHD (*p* < 0.001) groups compared to controls. In addition, a significant increase of ADMA concentrations was observed in patients belonging to the PT + CHD group when compared to PT patients (*p* = 0.003, Figure 1C).

The correlation analysis demonstrated a negative correlation between CoQ10 and NT concentrations (r = −0.289, *p* = 0.022). CoQ10 was also negatively correlated with dental variables such as PD (r = −0.313, *p* = 0.013), BOP (r = −0.139, *p* = 0.015), and PI (r = −0.309, *p* = 0.014). Instead, NT concentrations were positively correlated with ADMA levels (r = 0.233, *p* = 0.006), CRP (r = 0.249, *p* = 0.049), and dental variables (CAL: r = 0.455, *p* < 0.001; PD: r = 0.453, *p* < 0.001; BOP: r = 0.440, *p* < 0.001; PI: r = 0.423, *p* = 0.001). As previously demonstrated [14], ADMA levels were positively correlated with the dental variables (CAL: r = 0.418, *p* = 0.001; PD: r = 0.379, *p* = 0.002; BOP: r = 0.335, *p* < 0.007; PI: r = 0.340, *p* = 0.006) and CRP plasma concentrations (r = 0.508, *p* < 0.001).

**Figure 2:**
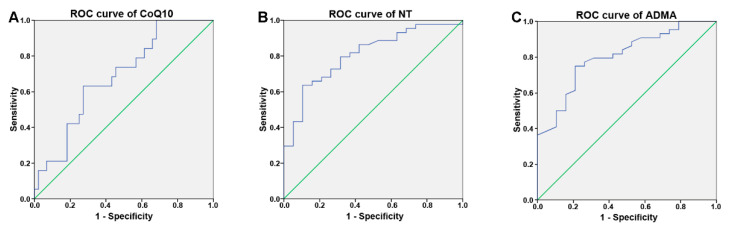
Receiver operating characteristic (ROC) curve for CoQ10 (**A**), NT (**B**), and ADMA (**C**) for their diagnostic performance in patients with PT and CHD (*n* = 44).

Receiver operating characteristic (ROC) curves were plotted to assess the diagnostic performance of CoQ10, NT, and ADMA in recruited patients (Figure 2). The area under the ROC curve (AUC) of CoQ10 was 0.684 (95% CI 0.548–0.820, *p*= 0.021, cut-off = 870 µg/L, sensitivity = 73%, specificity = 63%) (Figure 2A). NT showed an AUC of 0.807 (95% CI 0.605–0.919, *p* < 0.001, cut-off = 88 µg/L, 64% sensitivity, 89% specificity) (Figure 2B). ADMA produced an AUC value of 0.804 (95% CI = 0.691 to 0.916, *p* < 0.001, cut-off = 1.25 µmol/L, sensitivity = 75%, specificity = 79%) (Figure 2C). These data confirm that changes in the plasma concentrations of these compounds are associated with pathological conditions occurring in patients with PT and CHD when compared to healthy subjects.

The adjusted multivariate linear regression analysis aimed at assessing the possible influence of PT and CHD on biochemical markers. Our results demonstrate that CRP (B = 64,903, CI: 4.122–125.683, *p* = 0.037), PI (B = −20,129, CI: −38.848–−1.410, *p* = 0.036), and CHD (B = −358,692, CI: −626.926–−90.458, *p* = 0.01) were statistically significant predictor variables for CoQ10, while CRP (B = 0.132, CI: 0.065–0.181, *p* < 0.001), PI (B = 0.021, CI: 0.001–0.041, *p* = 0.044), and CHD (B = 0.499, CI: 0.300–0.698, *p* < 0.001) were statistically significant predictor variables for ADMA.

**Figure 3:**
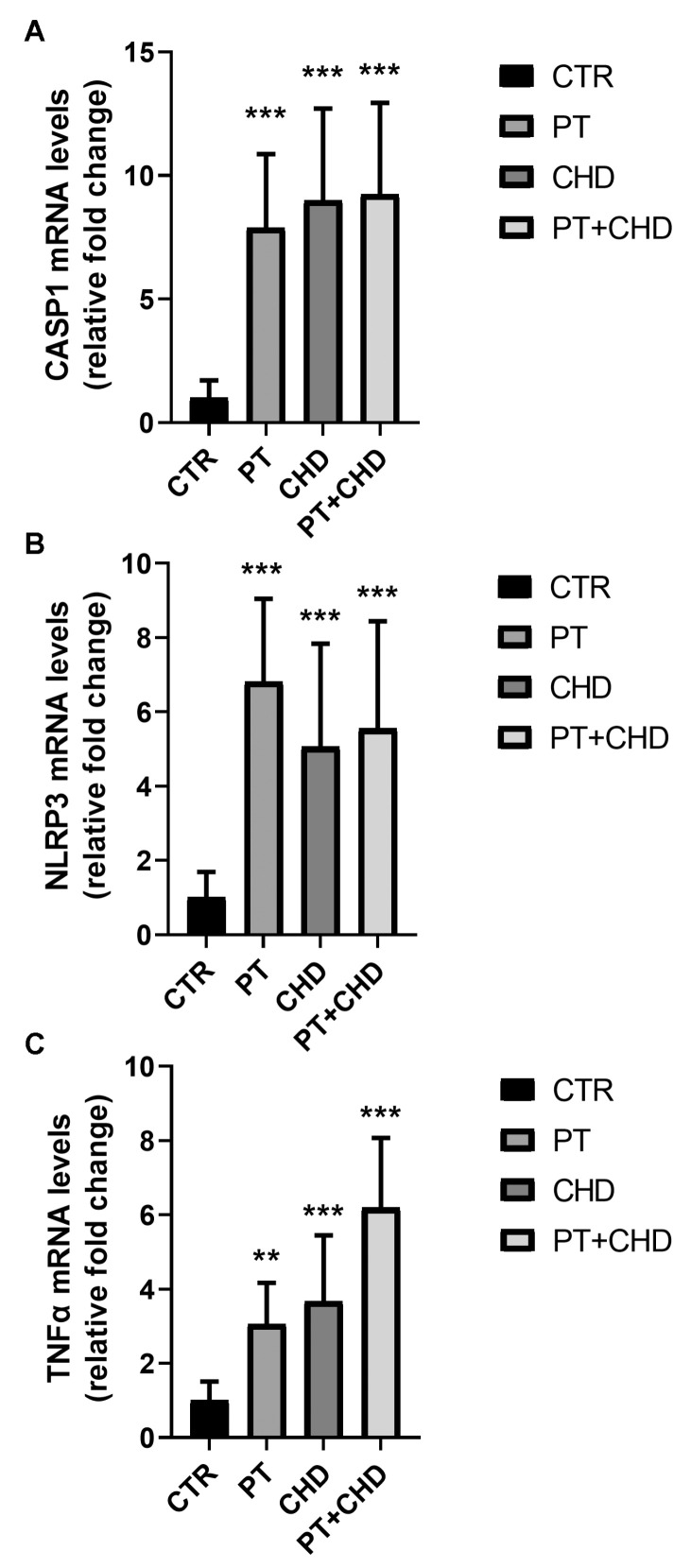
Analysis of CASP1 (**A**), NLRP3 (**B**), and TNFα (**C**) gene expression in PBMC from patients and healthy subjects. mRNA transcript levels were analyzed suing real-time PCR. The results are expressed as mean ± SD. ** *p* < 0.01 and *** *p* < 0.001 show significant differences in comparison to control subjects.

To ascertain the relationship between plasma biomarkers and the molecular changes in cell signalling associated with a pro-inflammatory status in PT and CHD, we evaluated the mRNA transcripts of the genes CASP1 and NLRP3, which are involved in the inflammasome pathway, in PBMC from patients and healthy subjects. We found an increase of inflammasome-related gene expression in PBMC from patients in comparison to the controls. The transcript levels of CASP1 and NLRP3 genes were up-regulated in all groups of patients when compared to healthy subjects (Figure 3A,B). Additionally, the mRNA levels of the TNF-α gene increased in patients in comparison to the control subjects, showing a significant increase in PT + CHD (Figure 3C).

## 4. Discussion

Many efforts have been made to describe the mechanisms underlying the cell response to oxidative stress occurring in CVD. The possibility of identifying new biomarkers has the additional potential for understanding a predictive diagnosis as well as the expected prognosis. A possible association between PT and CHD emphasizes the changes of some biomarkers such as ADMA, which is involved in endothelial cell dysfunction [18]. In a recent study, in subjects with CHD associated with PT, we observed that these pathological conditions are mainly associated with increases in ADMA plasma concentrations [14]. In the present study, subjects with PT + CHD also showed a significant increase in ADMA levels. In addition, in PT + CHD subjects, we observed an increased concentration of NT, a marker of nitrosative stress [19,20], associated with lower levels of CoQ10 in comparison to controls.

CoQ10 acts as an electron carrier in the mitochondrial electron transport chain, but it also controls the cellular redox state, plays a role in proton gradient formation in the endomembrane and the plasma membrane, and helps to control membrane structure and phospholipid status [21]. Its action as potent antioxidant at the level of cellular membranes and plasma by helping to recycle other antioxidants, such as vitamin C or vitamin E, and directly acting as a reducing and neutralizing agent of free radicals or oxidants has been well established [21]. Due to its roles, CoQ10 deficiency has been demonstrated to be involved in the pathogenesis of many degenerative conditions, including CVD [22]. Indeed, CoQ10 plasma levels have been found to be an independent predictor of mortality and prognosis in patients with heart failure, while its supplementation in these patients may have beneficial effects in improving symptoms and reducing major adverse cardiovascular events [23]. The antioxidant properties of CoQ10 can exert protective effects against hypertension and atherosclerosis by preserving NO synthesis, which is inhibited by oxidative stress and reducing LDL peroxidation and endothelial dysfunction [23].

On the basis of our results, we hypothesized an increase in the availability of superoxide oxygen anion. Then, it is possible to speculate that reduced CoQ10 levels and increased ADMA concentrations, causing a reduction of NO availability, with consequent peroxynitrite production, may be responsible for increases in protein nitration and NT production [24].

Protein nitration can have a remarkable impact on protein structure and function, and increased levels of NT have been found in different pathological conditions associated with oxidative stress status, such as CVD and neurodegenerative diseases, inflammation, and aging [25,26]. Additionally, NT has been suggested as a marker for atherosclerosis risk assessment. Indeed, the tyrosine nitration of proteins, including fibrinogen, apolipoprotein A-1, and apolipoprotein B-100, has been found in plasma from subjects with coronary artery disease, suggesting that changes in the function of some nitrotyrosine-modified proteins can create a pro-thrombotic and pro-atherosclerotic milieu [27]. The most important nitrosative compound is nitric oxide (NO), and the reduced bioavailability of this molecule is central in endothelial dysfunction and also in promoting arterial remodeling through several mechanisms [28]. Thus, the observed alterations here may be associated with endothelial dysfunction.

Other than statistical evaluation confirming the significant differences between the four groups of recruited subjects, the analysis of the ROC curves defined the diagnostic ability for the examined parameters. In particular, similar values for the area under the ROC curve for nitrotyrosine and ADMA were observed. The area under the ROC curve of combined PT, CHD, and PT + CHD confirmed that changes of CoQ10, ADMA, and NT are associated in the metabolic pathway involved in tissue damage under pathological conditions. Under our experimental conditions, we found that both ADMA and NT plasma levels had a good sensitivity and specificity, suggesting their reasonable potential as biomarkers of endothelial damage.

Larger sample sizes and future confirmatory studies are required for assessing the usefulness of these biomarkers in the evaluation of cardiovascular risk.

It is of interest that other observations showed the elevation of ADMA levels in different pathological conditions, including diabetes, atherosclerosis, and CVD [14,29]. The condition of ‘endothelial dysfunction’ has been linked to variations between vasodilating compounds and agents with proliferative properties [30].

The most important nitrosative compound is nitric oxide (NO). The reduced bioavailability of this molecule is central in endothelial dysfunction and also in promoting arterial remodeling through several mechanisms [28]. Several studies carried out on subjects displaying other vascular classical risk factors and including CVD, demonstrated a possible association between CHD and inflammation [31,32].

We demonstrated a positive correlation between the plasma levels of NT and ADMA, and also confirmed previous results showing a correlation between ADMA levels and CRP in all subjects [14]. It is then possible to hypothesize that inflammation status is associated with endothelial dysfunction. Although numerous biomarkers of inflammation and endothelial dysfunction have been studied, little evidence seems to substantiate this finding [33]. This might be the result of the heterogeneity of the recruited subjects and from differences in the chosen biomarkers that are reported in different studies. In fact, the levels of different substances that are separately evaluated are also associated with fibrinolysis, coagulation, inflammation, or the nitric oxide pathway in selected subjects [34].

The inclusion of all of those conditions, which are known to promote the pathogenic mechanisms that are associated with inflammatory response, should be further characterized by the evaluation of immune cell response. Therefore, in subjects with PT, CHD, and PT + CHD, we also evaluated possible increases in the production of the biomarkers of PBMC inflammation, which are known to be involved in the inflammatory response. The increase in the NLRP3 inflammasome promotes the pathogenesis of various inflammatory diseases [35]. Our data indicated that RNA transcripts of both NLRP3 and CASP1 are over-expressed in the PBMCs obtained from all patients in comparison to those from the controls. The activation pathway that is useful in promoting increases in the RNA transcripts of NLRP3 are unknown. However, some hypotheses have been described. According to reported assumptions, it is possible to confirm that uncontrolled endoplasmic reticulum stress, activating several ROS-producing processes, may be responsible for ROS level increases, which in turn induce NLRP3 activation [36].

Overall, we can hypothesize that oxidative stress produced by inflammatory processes occurring in PT patients leads to a depletion of antioxidant defenses, as confirmed by the reduction of CoQ10 plasma levels. In addition, ROS stimulates the synthesis and the accumulation of ADMA, which in turn induces ROS production through NOS enzyme uncoupling [37]. These alterations promote a condition of nitrosative stress, which is another well-known risk factor of CVD.

These outcomes suggest that high levels of both NT and ADMA may be involved in endothelial dysfunction associated with CHD and PT + CHD. This status is combined with the up-regulation of the NLRP3 inflammasome and may play a key role in the pathogenesis and progression of CHD in PT patients [18].

A limitation of the study involves the relatively small number of recruited patients. Therefore, a careful statistical model building has been performed to avoid the inclusion of too many variables in the regression models; consequently, it was not possible to evaluate the influence of comorbidities, for instance smoking and diabetes, on variations in CoQ10, NT, and ADMA levels.

In addition, further information, including the Gensini score, the evaluation of atherosclerotic plaque, and the assessment of cardiac function, could be useful to better characterize the link between the analyzed markers and the severity of CHD.

Further studies are necessary to confirm the possible causal link between ADMA, endothelial dysfunction, and inflammation. In parallel, clinical studies should be focused on selected groups of patients.

## Figures and Tables

**Table 1:** Primer sequences used for SYBR green real-time PCR.

Gene	Primer	Sequence 5′ → 3′
*β-ACT*	forward	TGGTTACAGGAAGTCCCTTGCC
	reverse	ATGCTATCACCTCCCCTGTGTG
*CASP1*	forward	GAACAAGGAAGAGATGGAGAA
	reverse	TCGGAATAACGGAGTCAATC
*NLRP3*	forward	GAAGACACCAGGACAATG
	reverse	GTCACCAAGAGGAACATC
*TNF-α*	forward	GTGAGGAGGACGAACATC
	reverse	GAGCCAGAAGAGGTTGAG

**Table 2:** Individual characteristics and biochemical parameters of recruited subjects.

	Controls (N = 19)	PT (N = 15)	CHD (N = 15)	PT + CHD (N = 14)
Age (years)	58.8 ± 11.6	62.8 ± 12.4	63.8 ± 9.7	68.4 ± 8.8
Sex—female, *n* (%)	7 (36.8)	9 (60)	5 (33.3)	9 (64.3)
Body mass index (kg/m^2^)	24.6 ± 2.4	24.9 ± 2.2	25.8 ± 2.1	25.5 ± 2.3
Fasting glucose (mg/dl)	112.4 ± 5.5	116.7 ± 7.4	118.4 ± 7.6	121.4 ± 8.5
Current smokers, *n* (%)	3 (15.8)	2 (13.3)	3 (20)	3 (21.5)
*Comorbidities*				
Diabetes, *n* (%)	-	3 (20) **	3 (20) **	2 (14.3) **
*Previous CVD*				
Atrial fibrillation, *n* (%)	-	-	4 (26.7) **^,§§^	6 (42.7) **^,§§^
Angina pectoris, *n* (%)	-	-	10 (66.7) **,^§§^	11 (78.6) **,^§§^
Stroke, *n* (%)	-	-	7 (46.7) **,^§§^	7 (50) **,^§§^
Heart failure, *n* (%)	-	-	7 (46.7) **,^§§^	6 (42.7) **,^§§^
*Drug treatment of CVD*				
Antihypertensive, *n* (%)	-	-	13 (86.7) **,^§§^	12 (85.7) **,^§§^
Statins, *n* (%)	-	-	9 (60) **,^§§^	7 (50) **,^§§^
Low-dose aspirin, *n* (%)	-	-	8 (53.3) **,^§§^	10 (71.4) **,^§§^
Beta blockers, *n* (%)	-	-	8 (53.3) **,^§§^	11 (78.6) **,^§§^
hs-CRP (mg/L)	2.6 ± 0.4	3.5 ± 0.6 *	7.1 ± 1.1 ***^,§§§^	6.7 ± 0.9 ***^,§§§^
VES	8.1 ± 1.6	13.5 ± 2.8 ***	11.7 ± 4.9 *	14 ± 3.8 ***
Total cholesterol (mg/dl)	183 ± 6.1	189 ± 6.7	204 ± 6.5	201 ± 6.8 *
Triglycerides (mg/dl)	184 ± 5.5	191 ± 5.9	195 ± 6.2	196 ± 6.6

Data are expressed as mean ± SD or number with percentage. * *p* < 0.05, ** *p* < 0.01, and *** *p* < 0.001 significant differences vs healthy subjects; ^§§^ *p* < 0.01 and ^§§§^ *p* < 0.001 significant differences vs PT patients calculated by the Mann–Whitney test. PT, periodontal disease; CHD, coronary heart disease; CVD, cardiovascular disease.

**Table 3:** Clinical dental variables of recruited subjects.

	Controls (N = 19)	PT (N = 15)	CHD (N = 15)	PT + CHD (N = 14)
N° of teeth	25 ± 0.9	18 ± 1.4 ***	22 ± 2.3 ***,^§§§^	17 ± 2.4 ***,###
CAL (mm)	1.3 ± 0.4	3.7 ± 0.3 ***	2.4 ± 0.5 ***,^§§§^	4.3 ± 0.6 ***,^§^,###
PD (mm)	1.4 ± 0.3	4.6 ± 0.5 ***	2.5 ± 0.5 ***,^§§§^	4.6 ± 0.7 ***,###
BOP (%)	9.1 ± 2.3	48.4 ± 5.2 ***	17.2 ± 2.7 ***,^§§§^	53.7 ± 5.7 ***,^§§§^,###
PI (%)	10.4 ± 1.5	35.6 ± 1.6 ***	15.1 ± 1.7 ***,^§§§^	33.0 ± 2.9 ***,###

Data are expressed as mean ± SD. CAL, clinical attachment level; PD, probing pocket depth; BOP, bleeding on probing; PI, Plaque index. *** *p* < 0.001 significant differences vs control subjects; § *p* < 0.05 and §§§ *p* < 0.001 significant differences vs PT patients; ### *p* < 0.001 significant differences vs CHD patients.

## Data Availability

The data presented in this study are available on request from the corresponding author. The data are not publicly available due to privacy restrictions.
